# Characterisation of a Live-Attenuated Rabies Virus Expressing a Secreted scFv for the Treatment of Rabies

**DOI:** 10.3390/v15081674

**Published:** 2023-07-31

**Authors:** Samuel P. Smith, Rebecca Shipley, Pascal Drake, Anthony R. Fooks, Julian Ma, Ashley C. Banyard

**Affiliations:** 1 Wildlife Zoonoses and Vector-Borne Diseases Research Group, Animal and Plant Health Agency (APHA), Weybridge, London KT15 3NB, UK; sam.smith@pirbright.ac.uk (S.P.S.); rebecca.shipley@apha.gov.uk (R.S.); tony.fooks@apha.gov.uk (A.R.F.); 2 Institute for Infection and Immunity, St. George’s Hospital Medical School, University of London, London SW17 0RE, UK; pdrake@sgul.ac.uk (P.D.); jma@sgul.ac.uk (J.M.)

**Keywords:** rabies, lyssavirus, virus attenuation, PEP, antibody, scFv, rabies treatment

## Abstract

Rabies virus (RABV) causes possibly the oldest disease and is responsible for an estimated >59,000 human fatalities/year. Post exposure prophylaxis (PEP), the administration of vaccine and rabies immunoglobulin, is a highly effective tool which is frequently unavailable in RABV endemic areas. Furthermore, due to the constraints of the blood-brain barrier, current PEP regimes are ineffective after the onset of clinical symptoms which invariably result in death. To circumvent this barrier, a live-attenuated recombinant RABV expressing a highly RABV-neutralising scFv antibody (62-71-3) linked to the fluorescent marker mCherry was designed. Once rescued, the resulting construct (named RABV-62scFv) was grown to high titres, its growth and cellular dissemination kinetics characterised, and the functionality of the recombinant 62-71-3 scFv assessed. Encouraging scFv production and subsequent virus neutralisation results demonstrate the potential for development of a therapeutic live-attenuated virus-based post-infection treatment (PIT) for RABV infection.

## 1. Introduction

Rabies, caused by all members of the lyssavirus genus, is a significant zoonosis affecting both human and animal populations globally with an estimated 59,000 human deaths annually [1,2,3]. The lyssavirus genus belongs to the Rhabdoviridae family of viruses, and as such, lyssaviruses contain a negative-sense single-stranded RNA genome encoding five viral genes: N (nucleoprotein), P (phosphoprotein), M (matrix), G (glycoprotein), and L (large polymerase) [4]. Rabies virus (RABV) is the prototypic virus of the lyssavirus genus with well-defined epidemiology in some areas for both volant and non-volant species. However, the epidemiology of the remaining 16 lyssaviruses (and one related but unclassified Kotalahti bat lyssavirus) [5] that have been most often reported from bats, remains largely undefined with often only a few isolates being available for each virus [6].

Rabies is an economically important zoonosis where its economic burden results from the treatment of illness, human deaths, and mass vaccination campaigns in multiple animal species [7]. These vaccination campaigns have been effective in eliminating reservoirs of dog-mediated rabies in multiple developed countries in Western Europe and the Americas [8,9]. However, in developing countries where rabies vaccination campaigns are not a priority, domestic dogs (*Canis lupus familiaris*) remain the primary reservoir [7]. Furthermore, because of the different cultural mentalities around responsible dog ownership and control, human infections occur [and, therefore, post-exposure prophylaxis (PEP) expenditure is required]. In this regard, Asia and Africa bear a disproportionate economic burden compared to developed countries, the majority of which comes from premature deaths, direct costs of PEP, and lost income while seeking PEP [3,7,10].

An effective immune response to RABV infection requires both B- and T-cell involvement [11,12,13] with gene knockout (ko) studies involving mice unable to generate an antibody response showing 100% mortality after attenuated virus challenge [14]. During the course of natural infection, anti-RABV antibodies [particularly of the immunoglobulin (Ig) G isotype] have frequently been found circulating in the blood and cerebrospinal fluid (CSF) of lethally infected animals and humans [15,16,17,18]. However, many studies have demonstrated that only small amounts of RABV-specific antibody cross the blood brain barrier (BBB) during pathogenic RABV infection. This is likely due to BBB permeability increasing prior to a peak in serum antibody titre [12,19,20]. However, while neutralising antibodies are vital in the clearance of RABV within the CNS, it is the production of high-affinity anti-RABV antibodies (which occurs during the peak of BBB permeabilisation) within the central nervous system (CNS) by infiltrating B cells which is the main contributor to viral clearance [12,14,21,22]. Lethal RABV [silver haired bat rabies virus (SHBRV)] has been shown to be cleared from infected mice upon the promotion of CNS inflammation to allow infiltration of antibody-producing B cells [12,19]. However, whilst the induction of CNS inflammation is inappropriate in the treatment of humans, this area provides an unfilled niche in clinical rabies treatment. Indeed, a therapy which is able to drive production of anti-RABV antibodies behind the BBB early in infection may be able to clear RABV from CNS tissues.

The RABV glycoprotein contains a number of antigenic sites thought critical for targeted neutralization [23]. In 2002, the World Health Organisation (WHO) proposed six RABV-neutralising monoclonal antibodies (mAbs) for investigation: 62-71-3, E559, 1112-1, D8, M777, and M727 [24,25,26]. Each of these mAbs targeted different antigenic sites (AS) within G. Of these (after the exclusion of 1112-1 and mAb D8 due to intellectual property conflicts and insufficient strain coverage, respectively), all targeted AS-II, with the exception of 62-71-3 which targets AS-I (with epitope mapping revealing specificity to amino acid residue K226 in the G protein) [25,27,28]. It was observed that 62-71-3 was expressed both as a single chain fragment variable molecule (ScFv) in a phage display system to investigate neutralisation and a chimeric murine–human antibody, which were later expressed in a ΔXF *Nicotiana benthamiana* plant expression system [25,29,30]. These were shown to be effective at neutralising phylogroup I lyssaviruses (such as RABV), but not phylogroup II or III lyssaviruses [31,32].

Since the development of the Milwaukee protocol (and the similar Recife protocol [33]), no major novel rabies treatment regimens or therapies have been approved for use in humans. However, as discussed previously, notable scientific advances have been made using live attenuated vaccine vectors (LAVVs) [34] and mAbs [35] for in vivo studies. As has been reviewed previously [34], in the context of post-symptomatic treatment, a large proportion of research has recently focused upon the attenuation of RABV by the insertion of NVEs or multiplication of attenuated RABV G genes [36,37]. These LAVVs have consistently been able to promote strong migration of T cells, B cells, and antigen presenting ells (APCs) into the brain and CNS. Where assessed, this has been accompanied by the permeabilisation of the BBB [34]. While the majority of novel LAVVs have been assessed as PrEP vaccination alternatives, some have been evaluated for their ability to prevent mortality if given as a post-infection treatment. LAVVs that have been investigated in this regard include TriGAS (also known as SPBAANGAS-GAS-GAS) [37], SRV9 [38,39], or a GM-CSF expressing LBNSE rabies virus vector [40]. These were all effective in preventing mortality in a minimum of 20% of mice lethally challenged peripherally with rabies virus. While these studies have not investigated the effectiveness of LAVV administration as a post-symptom onset treatment (only up to six days post-infection), they indicate that LAVVs represent a possible method for the post-clinical onset treatment of rabies. Another method, although notably more invasive than LAVV administration, is the intracerebroventricular administration of mAb cocktails directly onto the brain of lethally infected mice. This has been effective in successfully preventing mortality after lethal RABV infection [35]. This represents the greatest step towards an efficacious treatment of rabies. However, this technique is highly invasive, expensive, intensive, and relies on large volumes of mAb to be fully effective.

Here, the development of a novel post-infection treatment (PIT) for rabies is described. Specifically, this novel PIT uses a RABV-based LAVV to express the fluorescent secreted neutralising anti-RABV scFv molecule 62-71-3. By using a RABV-based LAVV, neutralising 62-71-3 was produced both in high concentrations (due to the ability of the LAVV to replicate in the host) and behind the BBB as the LAVV follows the typical route of RABV infection. Furthermore, this LAVV also activates the host immune system resulting in the clearance of both itself and any lethal RABVs present.

## 2. Materials and Methods

### 2.1. Mammalian Cell Culture 

Baby hamster kidney 21 (BHK-21), BSR-T7/5 (a BHK-21 cell line derivative), and neuronal-2a (N2a) cells were maintained in DMEM media (Fisher, 11574486) containing 10% FBS 10 U/mL penicillin and 10 µg/mL streptomycin. hCMEC/D3 BBB model cells (Merck, Darmstadt, Germany, SCC066) were maintained according to the manufacturer’s instructions in EndoGRO^TM^-MV Complete media in Collagen Type I rat tail (Merck, Darmstadt, Germany, 08-115)-coated T75 flasks.

### 2.2. Molecular Biology Techniques 

PCR was undertaken using NEB Q5^®^ DNA Polymerase (M0492L) according to the manufacturer’s instructions and products isolated using either NEB Monarch^®^ DNA Gel Extraction Kit (T1020L) or NEB Monarch^®^ PCR & DNA Cleanup Kit (T1030L). To generate cDNA from viral RNA extraction, the QIAGEN OneStep RT-PCR Kit was used (Qiagen, Hilden, Germany, 210212) according to the manufacturer’s instructions. DNA cloning using NEBuilder^®^ HiFi DNA Assembly was performed using the NEBuilder^®^ HiFi DNA Assembly Cloning Kit (NEB, Ipswich, MA, USA, E5520S) as per the manufacturer’s instructions. Site directed mutagenesis was undertaken using the Q5^®^ Site-Directed Mutagenesis Kit (E0554S, NEB) as per the manufacturer’s instructions. 

### 2.3. Rabies Virus Culture and Rescue 

Two RABV strains were used in this study. RABV-cSN (also known as SAD-B19, M31046.1) is an attenuated (when delivered peripherally) laboratory-adapted RABV strain. RV437 (KF154997.1) is a pathogenic RABV strain isolated from a racoon dog in Estonia. For RABV rescue, 18 h before transfection 3 × 10^4^ BSR-T7/5 cells were seeded per well in a 96-well plate (Corning, Amsterdam, The Netherlands). Per well, 40 ng of RABV-helper plasmids (pTIT-N, pTIT-P, pTIT-L [41]) and 200 ng of full-length RABV construct was simultaneously transfected using FuGENE^®^ 6 Transfection Reagent (Promega, Hampshire, UK) at a 3.5:1 ratio in 10 μL of total transfection mix. After incubation at 37 °C 5% CO_2_, cells were observed for fluorescence and passaged until 100% cellular infection was observed. To harvest the virus, supernatant was aspirated and cells were freeze-thawed three times before storage at −80 °C.

### 2.4. Virus Titration and Growth Kinetics 

The titre of cultured viruses was determined in focus-forming units per mL (FFU/mL) as described previously [42]. Briefly, BHK-21 cells were seeded and 10-fold serially diluted RABV was added in triplicate. After fixation with 80% acetone and staining with FITC anti-rabies monoclonal globulin (Fujirebio), an FFU/mL value was calculated.

To investigate virus growth kinetics, multistep growth curves were performed as described previously [43]. Briefly, BHK-21 cells were cultured in 12-well plates and RABV added at a moi of 0.01. After one hour, the cell monolayer was washed and media replaced. Media samples were taken at 0, 6, 12, 24, 48, 72, 96, and 120 h and immediately stored at −80 °C. After all samples had been taken and stored at −80 °C, virus titration was undertaken.

### 2.5. ELISA and Western Blot 

The Enzyme-Linked Immunosorbent Assay (ELISA) protocol used was based upon the Platelia^®^ Rabies II Kit (BioRad, Hertfordshire, UK) with some experimental modifications. These modifications included, after diluting samples in R6 buffer and incubation on pre-coated Platelia^®^ plates, a 1/1000 dilution of a rat anti-mCherry (5F8, chromotek) used as the primary antibody, and an anti-rat HRP-labelled antibody (BioLegend, San Diego, CA, USA, 405405) as the secondary. All OD450 values were expressed as a fold change from the negative media-only control. 

For western blot analysis, after samples were incubated with LDS sample buffer, separated on an SDS-PAGE gel, and transferred to a nitrocellulose blotting membrane, mCherry was visualised using an anti-red fluorescent protein antibody (Chromotek, Planegg-Martinsried, Germany 6G6) and a secondary anti-mouse horseradish peroxidase (HRP) antibody (Merck, Darmstadt, Germany, A2554). HRP was visualised using the ECL™ Prime Western Blotting System (Merck). 

### 2.6. Fluorescent Antibody Virus Neutralisation Test

Fluorescent antibody virus neutralisation (FAVN) [43] and modified (m)FAVN [31,44] assays were undertaken as described previously. Briefly, inactivated media from RABV-62scFv-infected cell culture or sera from animals were serially titrated across a 96-well plate. In total, 100 TCID50 of RABV CVS strain was then added to each well. After a one-hour incubation, BHK-21 cells were added to all wells to a final concentration of 3 × 10^4^ cells/well and incubated for a further 48 h. After acetone fixation and RABV N protein visualisation, the 50% endpoint dilution (where complete neutralisation appeared in 50% of wells) was calculated using the Spearman-Kärber method [45]. Antibody titre was expressed as International Units (IU) per mL or reciprocal titre.

### 2.7. Cell to Cell Spread 

The ability of RABVs to spread between cells was assessed by infecting a monolayer of either N2a or BHK-21 cells with a low concentration of virus particles and observing the number of cells per foci at various time points. BHK-21 cells were seeded and incubated overnight at 34 °C 5% CO_2_. Cells were then infected with 0.001 moi of virus for one hour at 37 °C 5% CO_2_. Cells were washed and a 0.3% agarose-GMEM overlay mix was gently added and allowed to set at room temperature, and cells were then incubated as before at 34 °C 5% CO_2_. At 24, 48, 72, 96, and 120 h post-infection, cells were fixed in 80% acetone and N protein visualised using a FITC-conjugated antibody (Fujireibio, Mölndal, Sweden). 

### 2.8. Cell Viability 

Assessment of the toxicity of virus infection was performed on N2a cells using the CellTiter 96^®^ AQueous One Solution Cell Proliferation Assay (MTS) (Promega, Hampshire, UK) according to the manufacturer’s instructions. Analysis of results was performed using the following formula and displayed as a percentage reduction of cell viability compared to uninfected cells.

### 2.9. hCMEC/D3 Cells

hCMEC/D3 cells were cultured according to the manufacturer’s instructions. After 12-well semi-permeable tissue culture membrane transwell inserts (3.0 µm pore size [46], Corning, Amsterdam, The Netherlands, 353181) were treated with rat tail collagen (Merck, 08-115) for one hour, 5 × 10^4^ hCMEC/D3 cells were seeded on each insert and incubated at 37 °C, 5% CO_2_. To assess the permeability of the hCMEC/D3 monolayers, Lucifer yellow (LY) (Merck) was added to the cells (apical side) and samples were taken from the basolateral side at 24 intervals as described in Zhao et al. [47]. These samples were then compared to a range of LY standards which were used to interpolate LY concentrations. To assess the ability of RABV-62scFv to cross the hCMEC/D3 BBB model, after the hCMEC/D3 monolayer was allowed to form a barrier, an infectious dose of RABV-62scFv was added to the apical compartment and incubated on a shaking incubator at 37 °C 5% CO_2_ (100 rpm) for one hour. Alongside this, an equivalent concentration of heat-inactivated RABV-62scFv was also incubated on hCMEC/D3 cells. After incubation, RABV-62scFv (live or inactivated) was removed from the cells which were then washed three times with PBS that was then replaced with growth media. After 48 h, samples taken from the basolateral side of the cell culture insert were investigated for the presence of viral RNA.

### 2.10. In Vivo Studies

All in vivo experimentation was undertaken in ACDP3/SAPO4 biocontainment facilities at the Animal and Plant Health Agency (APHA), Weybridge, UK, and complied following UK Home Office regulations under the Animals in Scientific Procedures Act (1986) and Home Office license PP9020679. Three-to-four-week-old female mice (Balb/c) were purchased from Charles River (Margate, UK). After random allocation into groups of four, mice were vaccinated or challenged with either virus or mock challenged with cell culture media by either the intracranial (ic) or intravenous (iv) routes. Mice were then observed twice daily for the development of clinical symptoms. Clinical signs and symptoms were scored 0–5 according to the following system: 0, no sign; 1, hunched body/ruffled fur; 2, limb twitching; 3, hindquarter paralysis; 4, progressive paralysis; 5, terminal recumbency/death). Mice were terminated upon reaching score 1 by cervical dislocation under terminal anaesthesia.

### 2.11. Fluorescent Antibody Test (FAT)

To visualise RABV N protein within the brains of mice, a small amount of brain tissue was removed from the hindbrain and placed on blotting paper. In this test, brain from a known-positive animal was included as a positive control. After two impressions were made on a glass microscope slide, samples were fixed in 100% acetone and RABV N protein visualised using a FITC-conjugated antibody (Fujirebio Mölndal, Sweden). 

### 2.12. Rabies Tissue Culture Isolation Test

The presence of live virus in mouse brain was assessed using the rabies tissue culture isolation test (RTCIT). Here, a 10% *w*/*v* homogenate was made of brain tissue in PBS. In total, 100 µL of brain homogenate was then added to N2a cells which had been plated out 30 min beforehand [4 × 10^4^ cells per well in 200 µL of GMEM]. Similar to FAT, brain tissue from a confirmed positive mouse was used as a positive control. N2a cells were incubated for 24 h at 37 °C before the media was replaced and incubated for a further four days. Here, media was removed before cells were fixed in acetone and then stained with an anti-RABV N protein FITC antibody (Fujirebio) and observed for the presence of apple-green perinuclear fluorescence.

### 2.13. Statistics 

Data sets were analysed for statistical significance using the GraphPad Prism software. All data sets were analysed for normal (gaussian) distribution and either a Student’s *t*-test, one-way analysis of variance (ANOVA), or Kruskal-Wallis test was selected as appropriate. For ANOVAs, the F test value was also reported. The cut-off for statistical significance was set at a *p* value of 0.05, with * ≤ 0.05, ** ≤ 0.01, *** ≤ 0.001, and **** ≤ 0.0001. 

## 3. Results

### 3.1. Molecular Cloning and RABV Virus Rescue

The 62-71-3 scFv expression cassette, containing both a human heavy-chain signal peptide and an mCherry fluorophore, was inserted into the RABV-cSN genome upstream of the RABV N gene. Transcriptional start and stop sequences were also included to flank the 62-71-3 scFv expression cassette. NEB HiFi assembly was used to insert these sequences. Binding of the 62-71-3 scFv to the RABV G protein is affected heavily by the presence of the lysine (K) at amino acid 226 [25]. Mutating this K to an arginine (R) was shown to significantly reduce the ability of 62-71-3 scFv to bind to the RABV G protein. Therefore, site-directed mutagenesis was used to introduce this mutation.

Upon the successful insertion of 62-71-3 scFv-mCherry into the RABV-cSN backbone and the RABV G K226R mutation, the resulting construct (named RABV-62scFv) was taken forward to virus rescue. Successful virus rescue was confirmed by the observation of enlarging fluorescent foci under UV illumination. Interestingly, in contrast to the positive control virus for virus rescue, fluorescence in RABV-62scFv-infected cells appeared more granular than diffuse, suggesting the packaging of 62-71-3 scFv mCherry within endocytic vesicles.

### 3.2. Cell Viability

**Figure 1:**
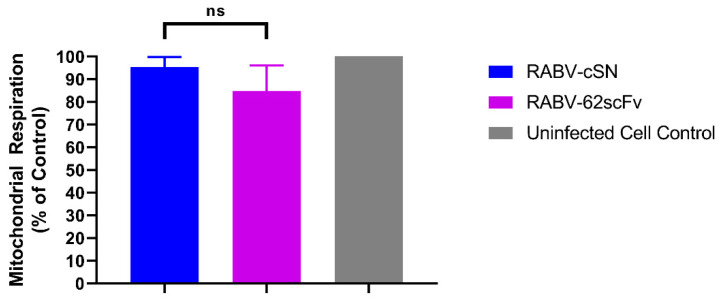
Assessing the effect of RABV-62scFv infection on mitochondrial respiration. An MTS assay was performed on N2a cells to assess the effect of RABV-62scFv and RABV-cSN infection on N2a cell viability. Output values were compared against the uninfected cell control to obtain a baseline value for mitochondrial respiration. The experiment was undertaken in triplicate to n = 3. Results were analysed using the Brown-Forsythe and Welch ANOVA Test (F* 5.299) where ns *p* > 0.05.

To investigate whether the expression of the 62-71-3 scFv mCherry polyprotein affected cell viability, mitochondrial respiration was investigated by MTS assay. N2a cells (mouse neuroblastoma origin) were selected as the cell line most relevant to the murine CNS. Furthermore, N2a cells have been shown to be highly sensitive to RABV infection [48,49], and NA cells (a subclone) have been used in previous studies when assessing the toxicity of novel recombinant RABV constructs [50]. No significant differences between cells infected with either RABV-cSN or RABV-62scFv were observed (Figure 1). 

### 3.3. Growth Kinetics

Growth kinetics of RABV-62scFv was assessed by multi-step growth curves in both BHK-21 and N2a cells alongside three other viruses: RABV-cSN, RBV-mCH, and RABV-K226R-mCH. Both BHK-21 and N2a cells were infected with either RABV-cSN, RABV-mCH, RABV-mCH-K226R, or RABV-62scFv and samples were taken at various timepoints. After the final sample collection, virus samples were titrated, and growth curve analysis undertaken.

**Figure 2:**
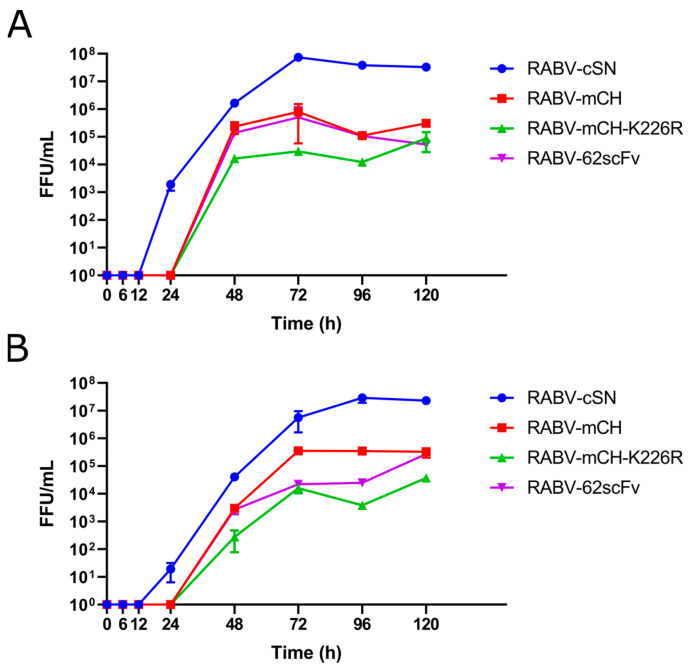
Growth kinetics of RABV-cSN, RABV-mCH, RABV-mCH-K226R, and RABV-62scFv. Growth kinetics were assessed by multi-step growth curves in both BHK-21 (**A**) and N2a cells (**B**). After 1 h infection of either a BHK-21 or N2a cell monolayer at 0.01 MOI, supernatant was taken at 0, 6, 12, 24, 48, 72, 96, and 120 h before FFU/mL was measured by virus titration. Experiment was performed in triplicate to n = 3.

In both BHK-21 and N2a cells, RABV-cSN was detectable after 24 h incubation. In BHK-21 cells, RABV-cSN plateaued from 72 h, while in N2a cells this was from 96 h. In both N2a and BHK-21 cells, RABV-mCH, RABV-mCH-K226R, and RABV-62scFv were present from the 48 h timepoint. In BHK-21 cells, all three virus titres plateaued at 48 h, while in N2a cells plateauing was only observed from 72 h (Figure 2).

The mean titre of each virus, upon plateauing, was evaluated for significant differences to compare virus growth between RABV-cSN, RABV-mCH, RABV-mCH-K226, and RABV-62scFv (Table 1). Statistical analysis showed significant differences between the post-plateau titres of RABV-cSN and RABV-mCH as well as RABV-mCH and RABV-mCH-K226R. This was consistent between growth curves performed in both BHK-21 and N2a cells. In BHK-21 cells, however, a significant difference in the post-plateau titres of RABV-mCH-K226R and RABV-62scFv was also observed. This significant difference was not present in growth curves performed in N2a cells.

The insertion of mCherry upstream of the N gene adversely affected RABV growth, as did the K226R mutation of the RABV G gene. Interestingly, the addition of the 62-71-3 scFv into the same open reading frame (ORF) as mCherry only affected growth kinetics when growth curves were performed in BHK-21 cells and not N2a cells. Furthermore, when comparing virus titres between N2a and BHK-21 cells, a significant difference was only observed in the titre of RABV-62scFv (*p* > 0.05, Brown-Forsythe and Welch ANOVA test).

### 3.4. Cell-Cell Spread

**Figure 3:**
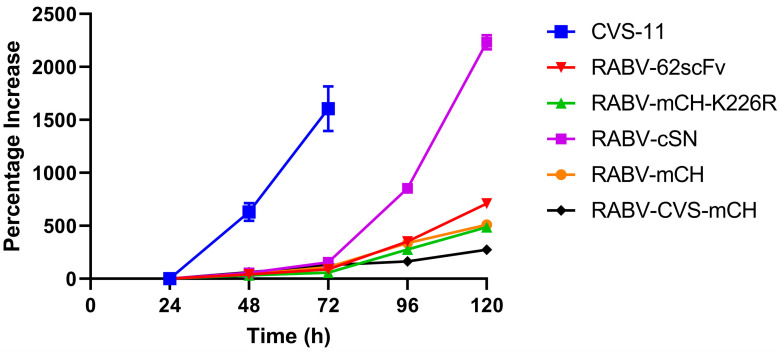
Viral dissemination assay investigating the differences between the cellular dissemination of RABV-cSN, RABV-mCH, RABV-mCH-K226R, RABV-62scFv, CVS-11, and RABV-CVS-mCH over a 120 h period.

To investigate the impact of both the RABV G gene K226R mutation and the insertion of the 62-71-3 scFv-mCherry cassette on the ability of RABV-62scFv to disseminate from cell to cell, an in vitro virus dissemination assay was developed. The kinetics of cell-to-cell viral dissemination of RABV-62scFv was compared against five viruses: RABV-cSN, RABV-mCH, RABV-mCH-K226R, RABV-CVS-mCherry, and CVS-11 allowing the effect of both the insertion of mCherry, 62-71-3 scFv, and the RABV G gene K226R mutation on cell-to-cell viral dissemination to be analysed. Furthermore, as cell-to-cell viral dissemination is indicative of virus pathogenicity, a comparison against known pathogenic viruses RABV-cSN, RABV-CVS-mCH, and CVS-11 was included to provide an indication of the potential pathogenicity of RABV-62scFv. Figure 3 shows the results of this experiment performed in N2a cells.

Pathogenic viruses RABV-cSN, RABV-CVS-mCH, and CVS-11 exhibited vastly different cell-to-cell viral dissemination patterns (Figure 3). While both RABV-cSN and CVS-11 displayed notably larger foci at earlier timepoints, RABV-CVS-mCH did not disseminate from cell-to-cell easily and foci size remained small throughout all timepoints. RABV-mCH, RABV-mCH-K226R, and RABV-62scFv foci size, similar to RABV-CVS-mCH, remained small throughout all timepoints assessed.

**Figure 4:**
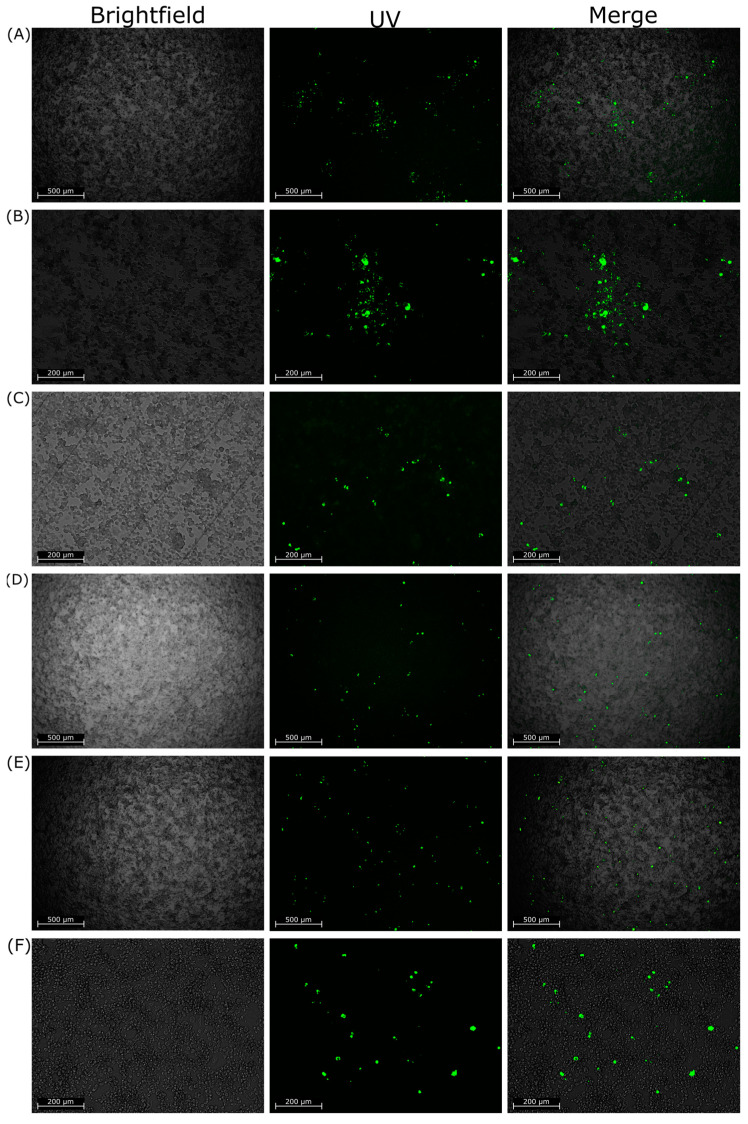
Representative images of foci generated in the cellular dissemination assay by either RABV-cSN, RABV-62scFv, or RABV-CVS-mCherry infection. (**A**,**B**) show images of foci generated by RABV-cSN infection ((**A**) at 10×, (**B**) at 4× magnification). (**C**,**D**) show images of foci generated by RABV-62scFv infection at similar magnifications, respectively. (**E**,**F**) show images of foci generated by RABV-CVS-mCH infection at similar magnifications, respectively. Images are displayed as brightfield, FITC fluorescence under UV, and a merged image. All cells have been stained for RABV N protein and visualised under UV. All images taken at 96 h post infection.

Interestingly, the phenotype of the foci generated by infection differed between the RABV viruses. The foci generated by RABV-cSN and CVS-11 infection were consistently concise and grew quickly. This contrasts with the foci generated by RABV-mCH, RABV-mCH-K226R, RABV-62scFv, and RABV-CVS-mCH infection. These viruses did not form concise foci and it became increasingly difficult to distinguish individual foci throughout later timepoints. Figure 4 shows representative images of foci formed by RABV-cSN (A/B), RABV-62scFv (C/D), and RABV-CVS-mCH (E/F) infection. As shown, RABV-cSN formed markedly larger foci than RABV-CVS-mCherry at equivalent time points.

### 3.5. Viral Expression of 62-71-3 scFv

To confirm the presence of recombinant 62-71-3 scFv in the extracellular media of RABV-62scFv-infected cells (termed r62-71-3 scFv), cell culture supernatant was removed and inactivated using beta-propiolactone (BPL). Appropriate controls were included such as inactivated media from cells infected with RABV-cSN, RABV-mCH, and RABV-K226R. Inactivated cell culture media was run on an SDS-PAGE gel before being analysed by immunoblotting by an anti-mCherry antibody (Supplementary Figure S1).

**Figure 5:**
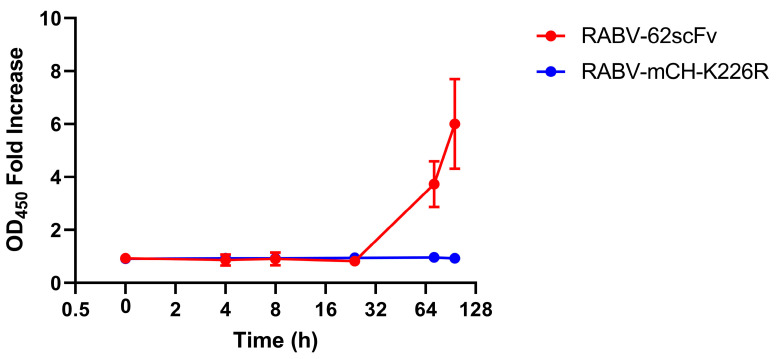
Charting r62-71-3 scFv production over time after BHK-21 cell culture monolayer infection with RABV-62scFv. Supernatant from RABV-mCH-K226R has been included as a control and results are shown as fold-increase of the negative media-only control. Where possible, error bars representing the standard error of the mean have been shown.

To assess the production of 62-71-3 scFv over time after BHK-21 cell culture infection, an ELISA was undertaken. This allowed the detection of 62-71-3 scFv in cell culture media. Overall, 62-71-3 scFv remained undetectable in the supernatant of infected cells until after 24 h, where exponential expansion was seen through the 72 and 96 h time points (Figure 5). To demonstrate that OD450 signal was due to 62-71-3 scFv binding, RABV-mCH-K226R was included as a control as this virus lacks the 62-71-3 scFv gene.

### 3.6. Neutralisation Properties of r62-71-3 scFv

A modified FAVN test was performed to assess the ability of cell culture media containing r62-71-3 scFv to neutralise RABV. Notably, r62-71-3 scFv was able to neutralise both CVS-11 and RV437 to equal extents, while supernatant from cells infected with RABV-mCH-K226R was unable to neutralise either (Table 2).

### 3.7. In Vitro Blood-Brain Barrier Model

**Figure 6:**
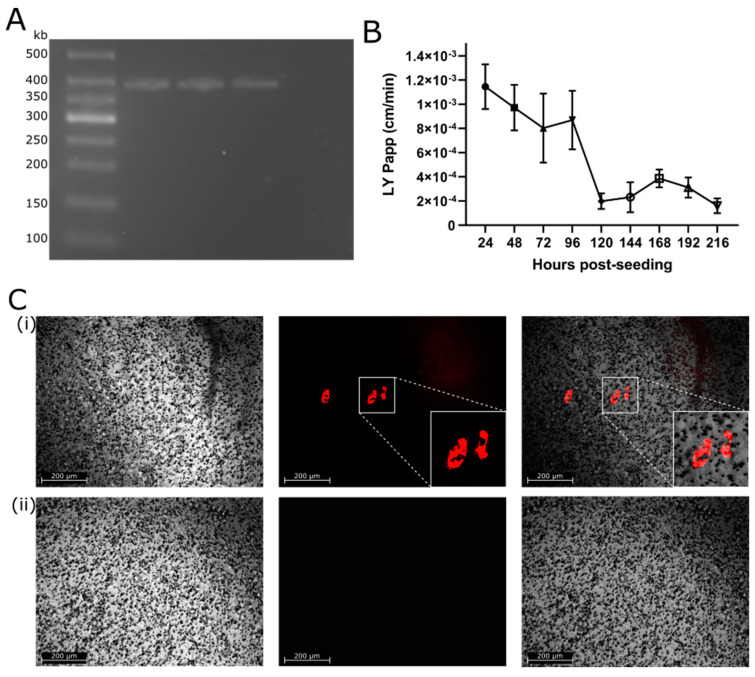
Assessment of the presence of RABV-62scFv to cross a hCMEC/D3 BBB. (**A**) samples taken from apical and basolateral compartments, respectively, after hCMEC/D3 monolayer infection (72 h) with RABV-62scFv (lanes 2/3) or heat-inactivated RABV-62scFv (lanes 4/5) and amplified for RABV-specific sequences. (**B**) Kinetics of hCMEC/D3 BBB model formation over time. At each 24 h time point after cell seeding, an apparent permeability (Papp) value was calculated. (**C**) Images taken of hCMEC/D3 blood-brain barrier monolayer to illustrate cellular infection with RABV-62scFv. Row (**i**) shows brightfield, UV illumination, and overlay images of hCMEC/D3 cells treated with live RABV-62scFv, while row (**ii**) shows images taken from hCMEC/D3 cells treated with heat-inactivated RABV-62scFv.

To assess the ability of RABV 62scFv to cross the BBB, we demonstrated that hCMEC/D3 cells formed an appropriate barrier 96 h after seeding (Figure 6A) and RABV-62scFv was shown to be able to cross this barrier (Figure 6B). As described previously, RABV-62scFv was added to the apical compartment and incubated for one hour on a shaking incubator. After 48 h, samples were taken from the basolateral side of the cell culture insert and RNA extracted. These samples were then assessed for the presence of viral RNA by RT-PCR.

To support the hypothesis that RABV RNA presence in the basolateral compartment was due to the infection of the hCMEC/D3 monolayer, images were taken of the hCMEC/D3 monolayer after RABV-62scFv infection under UV illumination. As shown in Figure 6C, red cell-localised fluorescence was observed, which is indicative of RABV-62scFv cell infection.

As shown in Figure 6, 72 h after infection RT-PCR detected RABV-scFv RNA in both the apical and basolateral compartments of the BBB model. In contrast, hCMEC/D3 monolayers that were treated with heat-inactivated RABV-62scFv revealed the presence of RNA in the apical compartment only.

### 3.8. In Vivo Toxicity Assessment of RABV-62scFv

Two routes of administration were used to assess RABV-62scFv in vivo toxicity: ic and iv. Here, Balb/c mice were infected with four different doses (10^1^, 10^2^, 10^3^, or 10^4^ FFU/dose) of either RABV-62scFv or a lethal positive control virus, RABV-CVS-mCH. A cell culture media control was included to control for complications of ic/iv inoculations. Mice were observed until the development of clinical symptoms where they were humanely terminated.

**Figure 7:**
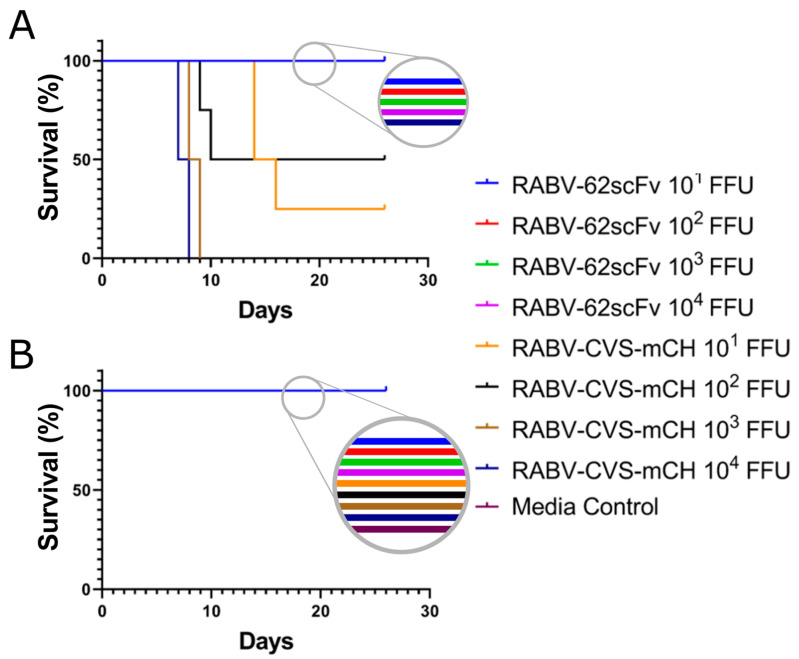
Survival curves of mice used to assess RABV-62scFv toxicity. Mice were given RABV-62scFv, RABV-CVS-mCH, or a media control either ic (**A**) or iv (**B**). Mice were then observed for 28 days for the development of clinical symptoms where they would be humanely sacrificed. Dosages of 10^1^, 10^2^, 10^3^, or 10^4^ FFU of virus (or media control) in 30 µL was delivered by needle either into the tail vein (iv) or brain (ic) of 3–4-week-old Balb/c mice.

As shown in Figure 7, as expected, all mice given the media control either ic or iv survived. Furthermore, when mice were given RABV-CVS-mCH ic (A), 100% mortality was observed at the doses of 10^3^–10^4^ FFU and 25% and 50% survival at doses 10^1^ and 10^2^ FFU, respectively. Therefore, as mortality after ic inoculation is indicative of successful infection of the mouse brain, these results demonstrate that the ic route was effective in delivering RABV directly to the brain. This was in contrast to mice delivered virus by the iv route. Regardless of dose, mice given RABV-CVS-mCH iv did not develop symptoms and all survived until the end of the experiment. Most importantly, regardless of the route of administration or dose given, all mice administered with RABV-62scFv survived until the end of the study and did not develop symptoms. In the case of iv administration, the lack of mortality may be due to an inability of RABV to properly initiate infection (as inferred by 100% survival in RABV-CVS-mCH-challenged mice). However, the presence of mortality in ic RABV-CVS-mCH-challenged mice indicated that the ic route was effective in allowing CNS and brain infection.

### 3.9. Serological Assessment of Mice Infected with RABV-62scFv

After humane sacrifice (either at the end of the study or at the development of clinical symptoms), sera were collected from the mice and a modified fluorescent antibody virus neutralisation test (mFAVN) was then performed to assess antibody titre.

**Figure 8:**
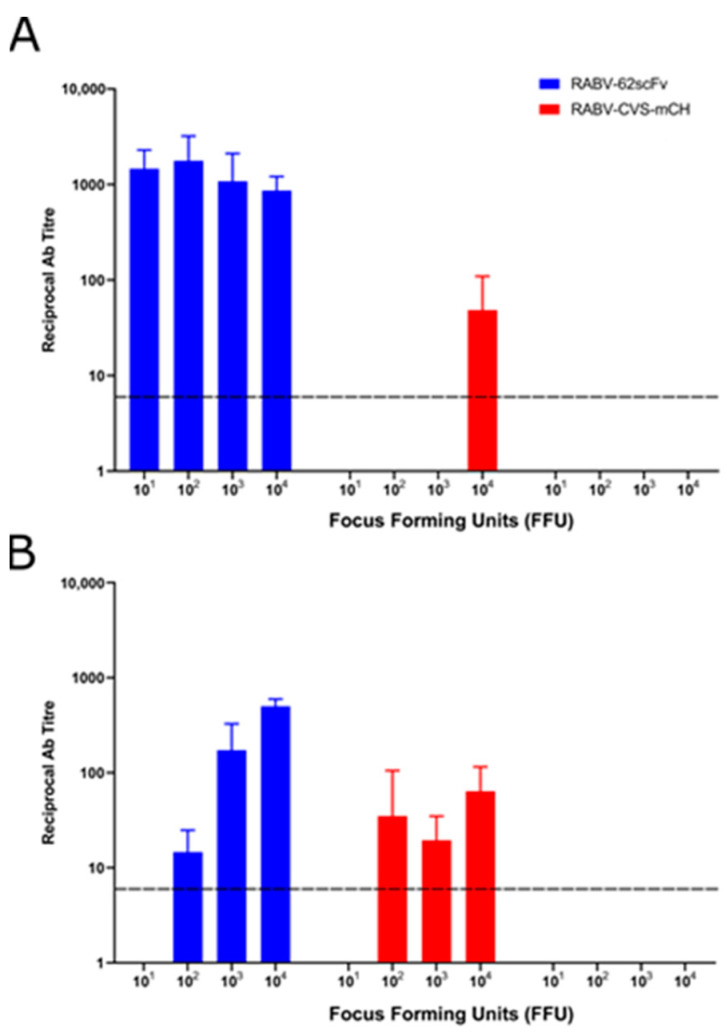
Serological assessment by mFAVN of mouse sera after challenge (ic or iv) at various FFU concentrations with either RABV-62scFv or RABV-CVS-mCH or media control. (**A**) shows results from mice challenged ic while (**B**) shows results from mice challenged iv. Sera from mice given a media control were tested for neutralisation against RABV-CVS-mCH. The dotted line represents the cutoff for detection at a reciprocal antibody titre of 7.49.

As shown (Figure 8), anti-RABV antibodies were present in high levels in all mice infected ic (A) with RABV-62scFv regardless of dose. Furthermore, only in mice given the highest titre of RABV-CVS-mCH ic were anti-RABV antibodies detected. When virus was delivered iv (B), the presence of anti-RABV antibodies correlated with the dose of RABV-62scFv given, with antibody levels below the cut-off for detection in mice given 10^1^ FFU RABV-62scFv. Antibodies were also present in mice given 10^2/3/4^ FFU of RABV-CVS-mCH; however, no correlation between dose given and anti-RABV antibody titre was observed. It is important to note, however, that RABV-62scFv or RABV-CVS-mCH was used to assess neutralising antibody levels in the sera of mice challenged with those viruses, respectively. This meant that, in the case of RABV-62scFv-challenged mice, only antibodies of host origin were measured and not scFv produced by RABV-62scFv cellular infection.

### 3.10. Investigating the Presence of RABV N Protein and Live Virus in Mouse Brain

To assess the ability of the host immune response to clear RABV virus from the brains of infected mice, after mice were humanely terminated (either at the development of symptoms or at the end of the study) a FAT and a RTCIT were performed on brain smears and brain homogenate to visualise RABV N protein and isolate live virus, respectively. Table 3 shows the results of these tests.

As shown in Table 3, regardless of group, dose, or challenge route, in all mice which developed symptoms of RABV, FAT and RTCIT revealed the presence of RABV N protein and live virus in brain smears and homogenate, respectively. Similarly, brains of mice which survived the duration of the experiment did not contain either RABV N protein or live virus.

### 3.11. Histopathological Assessment of CNS Immune Cell Infiltration

**Figure 9:**
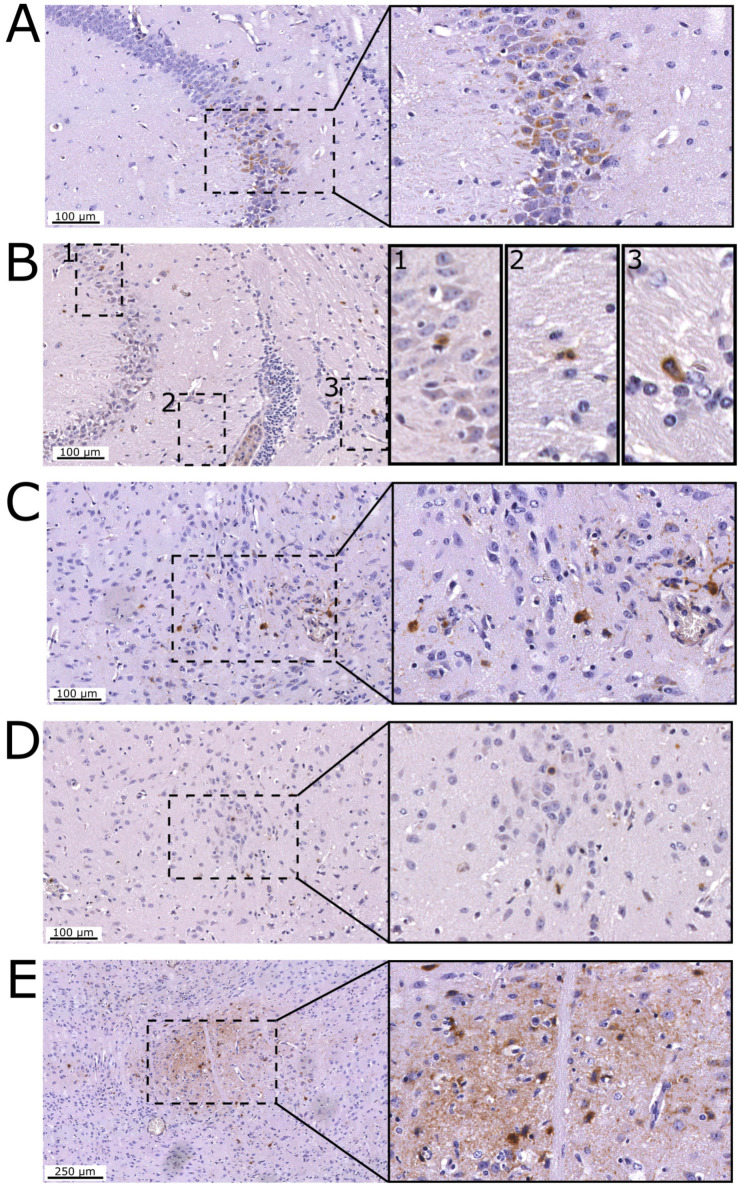
Histopathological assessment of mouse brains after ic challenge with RABV-CVS-mCH. Images (**A**–**E**) show representative images of sections from a mouse brain. Left hand side images have been taken at 200× magnification (**A**–**D**) or 100× magnification (**E**), with the right-hand side images showing magnification of the areas bordered by the dotted boxes in the left-hand side images at 400× resolution. (**A**,**B**) show the hippocampus region, where brown staining for the RABV N protein is shown in (**A**) and staining for CD3 is shown in (**B**). Images (**C**,**D**) show midbrain sections where brown staining indicates the presence of RABV N protein and CD3, respectively. Lastly, (**E**) shows a section of the thalamus where brown staining indicates RABV N staining.

The observations described previously suggest that, upon ic inoculation, RABV-62scFv was able to permeabilise the host BBB and activate the host immune system to allow clearance of RABV-62scFv from the brain and CNS. To investigate this, mouse brains were formalin fixed and paraffin embedded for histopathological investigation. Histopathological brain sections were taken from mice inoculated with RABV-CVS-mCH by the ic route. This was because mice inoculated with RABV-62scFv survived until the end of the experiment (Day 28) with no RABV N protein present (Table 3) in brain smears and would be absent of infiltrating immune cells. Sections of RABV-CVS-mCH-infected mouse brain were stained for RABV N protein and T cells (CD3). Images from these experiments are shown in Figure 9.

As shown in Figure 9A,B, within the hippocampus there were infrequent but focal areas of RABV N protein labelling. Furthermore, neighbouring areas were associated with rare CD3+ cells presence within the hippocampal laminar (1) and neuropil (2 and 3). In midbrain sections (C and D), scattered neuronal RABV N protein labelling was present with rare and dispersed CD3 presence. Lastly, while presence of CD3 was absent (image not shown), E shows a focal area within the thalamus with neuronal cells and neuropil labelled for RABV N protein.

## 4. Discussion

It is the case that 62-71-3 scFv was previously expressed in *N. benthamiana* plants using the pTRAK.6 plant expression vector [25,51] and was located within an expression cassette which allowed secretion into the extracellular environment. Here, to develop a system enabling the expression of the 62-71-3 scFv behind the BBB, this expression cassette was inserted into the genome of an attenuated RABV vector with a K226R mutation in the RABV G gene. This expression cassette was then inserted at position 1 within the RABV genome (upstream of the N gene) and flanked by gene borders taken from the RABV P gene. Initially, cell viability after infection was assessed using MTS assay, revealing that RABV-62scFv did not significantly affect cell viability compared to RABV-cSN. Growth kinetics of RABV-62scFv were then evaluated in both N2a and BHK-21 cells. After the construction of a series of viruses allowing the effect of each mutation to be investigated, growth kinetics revealed that the insertion of an additional ORF upstream of the N gene and the mutation of the G gene both had effects on viral growth kinetics. This revealed that the insertion of an additional ORF upstream of the N gene and the mutation of the G gene both had effects on viral growth kinetics. The insertion of an additional ORF upstream of the RABV N gene suggested a novel location for polymerase dissociation [52] to occur and subsequently all downstream proteins may be produced at lower levels. The impact of the RABV G gene K226R mutation on RABV growth kinetics is an observation that has been reported previously where mutational analyses of G have been undertaken [50,53]. Where assessed, impacted growth kinetics have been attributed to increased cellular apoptosis, lower G protein expression, inefficient G-M protein interactions [54], or decreased cell-to-cell viral dissemination. Of these, cell viability data indicated that impacted growth kinetics were not due to increased apoptosis, G protein expression was not assessed, and inefficient G-M interactions are unlikely to be involved as this occurs primarily at the cytoplasmic domain of G [54].

Cell-to-cell viral dissemination has been assessed in multiple other viruses and plaque morphology can be linked to altered phenotype [50,54,55,56,57,58,59,60]. In contrast to pathogenic RABV strains CVS-11 and RABV-cSN which demonstrated rapid plaque expansion with an exponential increase in the number of cells per foci, RABV-62scFv did not exhibit rapid cell-to-cell dissemination and foci rarely became larger than five cells. Interestingly, RABV-CVS-mCH displayed a similar pattern of cell dissemination to RABV-62scFv but not to CVS-11 or RABV-cSN, despite displaying in vivo pathogenicity following intracranial administration [43]. This may be due to the insertion of the mCH gene upstream of the N gene, similar to RABV-62scFv. Traditionally, the reduced dissemination of RABV-62scFv would suggest that RABV-62scFv is apathogenic upon in vivo infection; however, the reduced dissemination of RABV-CVS-mCH indicates that reduced cell-to-cell viral dissemination is not necessarily indicative of attenuation.

The proposed mechanism of action for RABV-62scFv involves the release of r62-71-3 scFv into the extracellular media of infected cells. Western blot was used to assess r62-71-3 scFv production from RABV-62scFv infected cells, with bands correlating to full-length r62-71-3 scFv in addition to an additional band that was present in the supernatant from cells infected with RABV-mCH, RABV-mCH-K226R, and RABV-62scFv. The size of this band correlated with monomeric mCherry and may be due to either the use of the RABV P genes transcriptional start and stop sequences [61] or post-translational cleavage in the (Gly4Ser)3 linker (which has been observed in previous studies [51]) and release upon cell death. Western blot analysis was also used to assess in vitro r62-71-3 scFv production over time, demonstrating an exponential increase in r62-71-3 scFv presence in cell culture media after 72 h. Furthermore, mFAVN experiments revealed that the r62-71-3 scFv produced after cell culture infection was effectively able to neutralise both CVS-11 and RV437 to similar extents. The ability of RABV-62scFv to cross the BBB is an important characteristic for RABV-62scFv to possess and this was assessed by the infection of an in vitro hCMEC/D3 BBB model. Here, it was shown that RABV-62scFv retained its ability to infect BBB cells, with viral RNA present in the basolateral compartment of the BBB model.

Upon in vivo investigation, RABV-62scFv was assessed for pathogenicity by two routes (iv and ic) at a range of concentrations. These routes were selected as ic represents the fastest route for delivery of RABV-62scFv-produced r62-71-3 scFv into the brain, while iv represents a route that would be favoured for therapeutic use in higher animal models due to minimal invasiveness and risk of complications. Results from the pathogenic control group indicated the iv route was ineffective for facilitating RABV infection. In contrast, delivery of RABV-62scFv by the ic route was effective in promoting a strong antibody response associated with clearance of RABV (both N protein and live virus) from the brain. Mice that were culled due to the onset of symptoms were also FAT- and RTCIT-positive and histopathological assessment of representative mice revealed the presence of RABV N protein (sporadically associated with CD3+ cells) within the midbrain, hippocampus, and thalamus regions of the brain. In summary, the in vivo data demonstrate that RABV-62scFv was apathogenic when delivered ic (which itself was shown to be an effective method of delivery of RABV directly to the brain) and resulted in the generation of a strong immune response, here characterised by the generation of neutralising antibodies, which allowed for clearance of RABV-62scFv from the brain.

In summary, the data presented in this manuscript have shown the design and assessment of a novel live-attenuated RABV which efficiently expresses the 62-71-3 scFv into the extracellular media which is able to both bind and neutralise infectious RABV and was continually produced over the course of in vitro cell infection. RABV-62scFv was then taken forward to in vivo assessment and found to be apathogenic upon ic inoculation.

## Figures and Tables

**Table 1:** Statistical differences between mean titres of RABV-cSN, RABV-mCH, RABV-mCH-K226R, and RABV-62scFv after plateauing in growth curves. A Brown-Forsythe and Welch ANOVA test was performed where *** *p* ≤ 0.001, ** *p* ≤ 0.01, * *p* ≤ 0.05, and ns *p* > 0.05. F* test values for BHK-21 and N2a statistical tests were 25.21 and 74.97, respectively.

	BHK-21		N2A	
Virus	Plateau Titre (FFU/mL)	Statistical Significance	Plateau Titre (FFU/mL)	Statistical Significance
RABV-cSN	4.83 × 10^7^	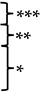	2.63 × 10^7^	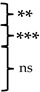
RABV-mCH RABV-mCH-K226R	2.51 × 10^5^ 2.96 × 10^4^		3.45 × 10^5^ 1.89 × 10^4^	
RABV-62scFv	8.76 × 10^5^		3.05 × 10^4^	

**Table 2:** Results of a FAVN test investigating the ability of r62-71-3 scFv to neutralise both CVS-11 RABV and RV437. As a control, supernatant from RABV-mCH-K226R-infected cells was used to assess CVS-11 neutralisation. Results are displayed as whether r62-71-3 scFv was neutralising or non-neutralising and are accompanied by an IU/mL or reciprocal titre value. The experiment was repeated three times and reciprocal titres averaged.

Challenge Virus	Media Assessed for Neutralisation	Neutralising/Non-Neutralising	Reciprocal Titre/IU/mL
**CVS-11**	r62-71-3	Neutralising	12.14/0.39
**CVS-11**	RABV-mCH-K226R supernatant sera	Non-Neutralising	n/a
**CVS-11**	Cell Culture Media Control	Non-Neutralising	n/a
**RV437**	r62-71-3	Neutralising	13.09/n/a
**RV437**	RABV-mCH-K226R supernatant sera	Non-Neutralising	n/a
**RV437**	Cell Culture Media Control	Non-Neutralising	n/a

**Table 3:** Description of the dose, challenge route, survival, presence of N protein, and live virus in mice used to assess RABV-62scFv toxicity. As results from FAT or RTCIT indicate either the presence or absence of RABV N protein or live RABV, results have been denoted with either a + (positive) or − (negative).

Virus Challenge Group	Dose (FFU)	Challenge Route	Survival to the End of the Study	Presence of RABV N Protein in Brain Smear (FAT)	Presence of Live Virus in Brain Homogenate (RTCIT)
**RABV-62scFv**	10^1^–10^4^	ic	Y	−	−
**RABV-CVS-mCH**	10^1^		N N N Y	+ + + −	+ + + −
**RABV-CVS-mCH**	10^2^		Y Y N N	− − + +	− − + +
	10^3^–10^4^		N	+	+
**RABV-62scFv**	10^1^–10^4^	iv	Y	−	−
**RABV-CVS-mCH**	10^1^–10^4^		Y	−	−
**Media Control**	N/A	ic/iv	Y	−	−

## Data Availability

Not applicable.

## Supplementary Materials

The following supporting information can be downloaded at: https://www.mdpi.com/article/10.3390/v15081674/s1, Figure S1: Western Blot of supernatant from infected cell culture.
