# Supplementary material for: Characterisation of a Live-Attenuated Rabies Virus Expressing a Secreted scFv for the Treatment of Rabies
Source: Viruses. 2023 Jul 31;15(8):1674. doi: 10.3390/v15081674 (PMC10458464; doi:10.3390/v15081674)
Supplement: Supplementary file 1 [file viruses-15-01674-s001.zip › viruses-2505922-supplementary.pdf]

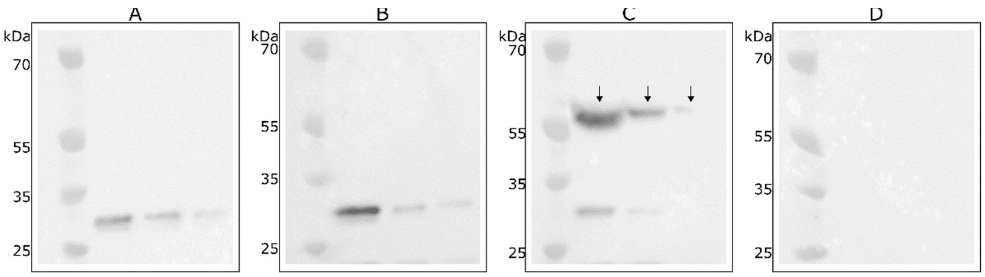

Supplementary Figure S1: Western Blot of supernatant from infected cell culture. Cells were infected with either RABV-mCH (A), RABV-mCH-K226R (B), RABV-62scFv (C), or RABV-cSN (D). After infection, any virus present in the supernatant was inactivated by BPL treatment. Supernatant was titrated in doubling dilutions and all dilutions ran on an SDS-PAGE gel before transfer to a nitrocellulose membrane. To visualise bands, nitrocellulose membranes were stained with an anti-mCHERRY (6G6, chromotek) antibody.
